# ITD sensitivity to naturalistic sounds in the superior olivary complex

**DOI:** 10.1186/1471-2202-12-S1-P216

**Published:** 2011-07-18

**Authors:** Michiel Remme, Jason Mikiel-Hunter, Roberta Donato, John Rinzel, David McAlpine

**Affiliations:** 1 Center for Neural Science, New York University, New York, NY 10003, USA; 2 Ear Institute, University College London, London, WC1X 8EE, UK; 3 Courant Institute of Mathematical Sciences, New York University, New York, NY 10003, USA

## 

Neurons in the medial superior olive (MSO) and lateral superior olive (LSO) of the auditory brainstem code for sound source location in the horizontal plane by extracting interaural time differences (ITD) from the fine structure or envelope of sound stimuli. Both cell types are tuned to frequency (characteristic frequency, CF) and are organized along a tonotopic axis.

The statistics of natural sounds vary with frequency, e.g., the signal to noise ratio of combined behaviorally relevant and background noise stimuli typically decrease with increasing frequency [1]. Also, auditory nerve encoding of sound changes with increasing frequency, moving from a phase-locking to an envelope coding strategy.

We studied whether the intrinsic properties of MSO and LSO cells vary along the tonotopic axis in order to optimize ITD sensitivity to natural sounds. Using *in vitro* whole-cell recordings we characterized the membrane filters of cells in the guinea pig MSO and LSO with ZAP current injections. All MSO cells and some of the LSO cells showed membrane potential resonances with peak frequencies between 80 and 400 Hz. The experiments suggest that the peak resonant frequencies decrease along the tonotopic axis (with increasing CF). Using a modeling approach we first assessed what membrane currents could underlie the resonance. Linear models fitted to the data predict that a gradient in both the density and activation time constant of a low threshold potassium current (I_KLT_) is probably underlying the resonant frequency gradient. We subsequently examined how the filter gradient affects ITD sensitivity to natural sounds. Simulations where we fed guinea pig vocalizations to the neural filters via an auditory nerve model show that ITD sensitivity increases with the cell’s peak resonant frequency, also in noisy environments. Hence, our results suggest that ITD sensitivity decreases along the tonotopic axis. This finding could underlie the decreasing performance in ITD discrimination with increasing CF and amplitude modulation frequency found in psychophysics [2].
